# Common factors in HIV/AIDS prevention success: lessons from Thailand

**DOI:** 10.1186/s12913-022-08786-6

**Published:** 2022-12-06

**Authors:** Joseph Harris, Suriwan Thaiprayoon

**Affiliations:** 1 Department of Sociology, Boston University, 100 Cummington Mall, Room 260, Boston, MA 02215 USA; 2 Division of Global Health, Ministry of Public Health, Nonthaburi, 11000 Thailand

**Keywords:** HIV/AIDS, HIV prevention, Thailand, Policy entrepreneur, Evidence, Demonstration project, Political opportunity, Intervention, Implementation, Qualitative

## Abstract

**Background:**

Thailand has achieved global acclaim for its response to HIV/AIDS. However, the success of some of the country’s most well-known initiatives was by no means a foregone conclusion. Policy entrepreneurs on the periphery of power had to achieve buy-in from stakeholders in state and society to scale and mainstream their ideas. This paper offers a comparative and historical understanding the process by which three of the country’s most well-known initiatives came into being: a civil society campaign to promote condom usage; a Ministry of Public Health program that aimed to prevent the spread of Human Immunodeficiency Virus (HIV) by targeting high-risk populations (the 100% condom program); and a universal Prevention of Mother-To-Child Transmission (PMTCT) program.

**Methods:**

The research relied on existing literature and interviews with high-ranking ministerial officials, representatives from international and non-governmental organizations, professors, and philanthropic organizations, in addition to a review of the existing literature. Taking a comparative and historical approach that is common within political science and sociology, we analysed the in-depth qualitative interviews in relation to the literatures and used an inductive cross-case analysis aimed to draw out critical features that the initiatives shared in common.

**Results:**

Common factors in HIV/AIDS prevention that cut across the three key cases include policy entrepreneurs who championed the programs, successful demonstration projects that produced a credible evidence base for policy adoption, and a diverse set of institutional partners that played critical roles in helping to mainstream their initiatives into national HIV/AIDS policy and scale programs nationally. The findings from this comparative research project have implications not only for the building of understanding related to one single project, but for broader theoretical understanding related to the mainstreaming of health policy from peripheral spaces of power.

**Conclusions:**

This analysis draws out the role that demonstration projects played in building a credible evidence base for policy adoption and the role that a diverse set of institutional partners played in elevating the profile of policy entrepreneurs’ ideas and helping to scale them nationally as state policy. Success was contingent on entrepreneurs first identifying and then taking advantage of different political opportunities that arose during each of the historical periods. Over time, these initiatives have evolved from vertical programs into an integrated program, in parallel with the evolution of the HIV/AIDS landscape at the global level.

**Supplementary Information:**

The online version contains supplementary material available at 10.1186/s12913-022-08786-6.

## Background

Thailand’s innovative Human Immunodeficiency Virus/Acquired Immunodeficiency Syndrome (HIV/AIDS) prevention policies have earned international recognition. In the 1980s, as the HIV/AIDS crisis was just emerging, the founder of a non-governmental organization, the Population and Development Association, sought to create more openness to condom use in Thai society by ‘thinking out of the box,’ holding condom-blowing competitions and making condoms more widely available [1]; staging a Miss Condom Beauty Contest and encouraging frequenters of go-go bars to use condoms [2]; and founding Cabbages and Condoms, a restaurant in Bangkok’s red-light district that used humor to overcome AIDS related stigma and condom use [3]. His efforts garnered the Gates Award for Global Health.

Thailand’s 100% condom program (or 100% condom use program) was implemented nationally in 1991-1992 and built on successful program that had been piloted in the province of Ratchaburi by the director of the region’s Communicable Disease Control Office. The pilot project had led sexually transmitted diseases (STDs) to fall from 13% prevalence to under 1 % in just 2 months [4]. The program sought to control spread by reducing transmission among sex workers and their clients, with a scaled-up national version of program resting on the annual distribution of 60 million free condoms [5], resulting in a 95% reduction in new infections [6]. The UN recognized Thailand’s comprehensive nation-wide HIV/AIDS prevention program as the first in Asia [7] and was featured within Joint United Nations Programme on HIV/AIDS (UNAIDS) Best Practice Collection [8].

In the mid-1990s, the prevalence of HIV among pregnant women in Thailand stood at 2 %, with transmission from pregnant mother-to-child standing at a rate greater than 20%; by 2015, prevalence dropped to 0.6% and transmission to 1.9% [9]. The introduction of a national Prevention of Mother-To-Child Transmission (PMTCT) program in 1999 proved highly effective in bringing about this transition, using zidovudine initially but later finding supplementation with nevirapine to be even more efficacious [10]. Thailand’s PMTCT program reached 98% of mothers living with HIV [11]. The World Health Organization (WHO) certified Thailand’s elimination of mother-to-child transmission of both HIV and syphilis in June 2016, making Thailand only the second non-Organization of Economic Cooperation and Development (OECD) country outside of Cuba to meet these marks [12] and the first country in Asia to do so [13].

By some estimates, Thailand’s HIV prevention programs collectively led seven to eight million new infections were averted [14]. However, these prevention programs had diverse institutional foundations and were respectively pioneered by the leader of a civil society organization, a health official in a province outside of Bangkok responsible for regional communicable disease control, and non-profit health researcher at the Thai Red Cross. However, in all cases, there are advocates who used the resources available to them to advance HIV/AIDS prevention policy, so-called “policy entrepreneurs,” who occupied positions on the periphery of state power with few levers available to them to make change nationally. How did these policy entrepreneurs overcome such challenges? What, if any, common strategies did they draw on to institutionalize policies at the national level?

## Methods

This study follows in the tradition of research on the health policy process [15, 16] and emerging work in political science and sociology [17–19]. An important body of work within this vein of scholarship has used inductive analysis of comparative cases to develop hypotheses and build theory [20, 21]. Analysis in the present study likewise uses inductive comparative analysis of cases to help build theory related to the institutionalization of policy innovation. We examine three different HIV prevention initiatives in Thailand: non-governmental organization (NGO)-led condom promotion efforts in the 1980s; the government’s 100% condom program in the early 1990s; and a national PMTCT program that developed through productive tensions between the Red Cross and the Ministry of Public Health in the late 1990s/early 2000s. In all three cases, policy entrepreneurs initially had to navigate the challenges of policy adoption, implementation, and sustainability from peripheral spaces outside the halls of power in the state capital.

This project was part of a broader project on Thailand’s contributions to global health that involved nearly 70 in-depth interviews with the most relevant ministerial officials, representatives from international and non-governmental organizations, professors, and philanthropic organizations, including policy entrepreneurs themselves and analysis of the existing literature. Twenty of these interviews bore on the topic of HIV/AIDS. Interviews were semi-structured and transcribed and ranged in duration from approximately 30 minutes to more than 2 h. Participants provided informed consent, and the study received human subjects approval from the Institutional Review Boards at Boston University and Thailand’s Ministry of Public Health.

The results of the analysis of each policy initiative are presented in turn, followed by discussion of the collective findings from all three areas. The findings offer lessons for policymakers and contribute to scholarly understanding of the causal mechanisms underlying policy change by means of tracing historical processes [22].

### Condom promotion in the 1980s

The highly visible role Thailand became known for in promoting condoms to prevent against HIV/AIDS had its roots in earlier family planning efforts in the late 1970’s and early 1980’s, driven by civil society aimed at curbing population growth. While tensions existed between government and civil society amid the transition from General Kriangsak Chamanan’s administration (1977-80) to government by Prem Tinsulanonda (1980-89), non-governmental organizations nevertheless played important, if somewhat circumscribed, roles in society contributing to concrete socioeconomic development efforts. Major donors, like the U.S. Agency for International Development, sponsored condom promotion programs in Thailand and other countries during this time [23, 24], and the Population and Development Association (PDA) emerged as one of Thailand’s most important non-governmental organizations in the field family planning through its extensive condom distribution efforts. These efforts complemented high levels of contraceptive coverage (between 94 to 98%) provided by the Ministry of Public Health (MOPH) at a time when condom usage in the country remained very low (between two and 4 %) [25].

Whereas in 1960 the population growth rate was 3.3% and just 3% of eligible Thai couples used some form of contraception, by 1984 the population growth rate had reduced by more than half, and 65% were doing so [2]. PDA did this by bringing contraceptive technologies beyond the doctors’ office and enlisted community members to distribute contraceptive technologies to the people. By 1980, PDA had 10,800 distributors operating in 16,000 villages and 48 provinces [2, 26].

A signature aspect of PDA’s approach involved using humor to open space for people to talk about sex and condoms. In one early training session for 1000 teachers, PDA founder Mechai Viravaidya broke the stigma related to talking about condoms by blowing up condoms and telling jokes [27]. Over the course of 5 years, PDA trained some 320,000 rural school teachers, who helped deliver the message to the children and students they taught [27]. This early work demonstrating results in family planning helped paved the way for the use of similar prevention efforts when the country confronted HIV [28].

With the advent of the AIDS epidemic in the mid-1980s in Thailand, PDA started an AIDS public education campaign in 1987 [2]. PDA brought the message that condoms could save lives to Patpong, the center of Thailand’s urban sex industry, creating a Miss Condom Beauty Contest and encouraging frequenters of go-go bars to use condoms [2]. The humor communication strategy for condom use was effective in tackling the issues deemed culturally sensitive in Thailand. Aside from the condom-blowing contests in Patpong, the PDA founder started Cabbages and Condoms, a restaurant in Bangkok’s red-light district that used humor to overcome AIDS related stigma and condom use [3]. This work aimed to involve everyone from gas stations to McDonald’s, from business to religious actors in the fight against AIDS [27]. PDA sought to recognize the relationship between rural poverty and HIV/AIDS, with poor families sending young people to the city to work, who would often return home jobless with HIV [23] and address the needs of villagers by developing sustainable economic opportunities for rural populations [29].

In the mid-1980s and early 1990s, the notoriety that the PDA founder had developed from starting PDA enabled him to transition to an insider status in government, becoming a senator and minister. These new positions provided him further opportunities as a policy entrepreneur to mainstream HIV/AIDS prevention efforts within government. When Thailand’s newly elected Prime Minister initially proved difficult to enlist in the fight against AIDS following elections in 1989, he found key institutional partners in the military and offered a briefing which led the military to take action on the issue [2]. He sought to heighten the visibility of the AIDS epidemic in Thailand, through a study that included projections of four million HIV infections by 2000, 25 years of productive work lost per person, and one-fifth of national GDP lost to AIDS [28]. (By 1994, HIV prevalence was 4 % among army recruits alone [6], including 12% in the Upper North [5].) After the military formed the National Peace Keeping Council and appointed Anand Panyarachun to act as interim Prime Minister, the policy entrepreneur found a new institutional partner in the Prime Minister’s Office, which appointed him to be Minister for the Prime Minister’s Office, overseeing such issues as tourism, information, and AIDS. His continued advocacy in this role played an important role in helping to convince politicians of the need for multisectoral cooperation and coordination [23].

While these efforts helped to increase awareness of HIV/AIDS and to heighten the visibility of condoms in Thailand, it is important to acknowledge that these efforts alone do not account for Thailand’s success in combatting HIV/AIDS. Indeed, cases of HIV/AIDS grew by over 150,000 in spite of the mass public health campaigns that occurred before and during the epidemic [25]. Strong surveillance programs by MOPH were clearly an important precondition for the country’s overall success [25], as well as other HIV/AIDS prevention programs, including the two that will be surveyed in the remainder of the paper.

Although PDA’s initial family planning activities were circumscribed in nature, the organization’s success enlisting citizen participation to confront stigma by opening conversation space about sensitive issues built a credible platform for further mainstreaming of HIV/AIDS prevention efforts within government when the AIDS crisis emerged and political opportunities arose to make change from within government. High impact research and strong advocacy helped turn military officials into partners in the fight against HIV/AIDS and the Prime Minister in a caretaker government to take more aggressive action. Similar multi-sectoral, community-focused approaches that were patterned after these early approaches later featured in the UNAIDS Best Practices Collection [30].

### The 100% condom program

Thailand’s HIV/AIDS epidemic was initially concentrated heavily in the country’s illegal but highly visible commercial sex industry, with more than 80% of infections found among sex workers and their clients [31]. By the late 1980s and early 1990s, almost daily funerals among sex workers in the epidemic’s center of Chiang Mai in northern Thailand, fostered a growing awareness of the gravity of the situation. An estimated 44% of sex workers in Chiang Mai were HIV positive in 1989 [32]. By 1994, HIV prevalence among sex workers rose to 31% nationally [5].

Given the high prevalence in that particular sector, a policy entrepreneur within the Ministry of Public Health – and the first director of Thailand’s Center for the Prevention and Control of AIDS in 1987 – theorized that if the country could reduce infection among sex workers, it could control HIV infection more broadly [33]. International approaches to the prevention of sexually transmitted infections at the time sought to motivate people to seek diagnosis and treatment [6]. Ordinary educational programs and programs that aimed to reduce or prevent sexually transmitted infection alone would not be enough [34]. What was needed was a way to ensure that sex workers used condoms 100% of the time. Working with a key institutional partner at the provincial level in the provincial governor, he set up a pilot project with this aim in the province of Ratchaburi in 1989. The program threatened closure of commercial sex work establishments if condoms were not used, using STI screening to track where infections were occurring [34]. The pilot program led not just HIV infections to fall but sexually transmitted infections more generally to fall from 13% prevalence to under 1 % in just 2 months [4].

However, the policy was not uncontroversial: although prevalent, commercial sex work was illegal, and there were pitched debates at the time about whether government funds should be used to subsidize unfaithful husbands’ activities [23]. Other alternative approaches at the time emphasized targeting men’s behavior and creating occupational training programs that aimed to incentivize sex workers to change their occupation [34]. Making matters more complicated, without national support for the policy, sharing of the Ratchaburi experience through bilateral visits to different provinces meant the program might take 6 years or more to scale nationally, so a way had to be found to for the policy to be adopted at the national level by the Ministry of Public Health [34]. Initial efforts to build support for the program through formal channels within the Ministry’s Communicable Disease Control regional offices were not successful, and the program received only moral support from an influential but informal group of MOPH affiliated executives concerned with advancing health equity, who expressed concern about its feasibility [6, 34].^1^

This policy entrepreneur eventually found support from a key institutional partner in the Permanent Secretary, the highest position held by a civil servant in the ministry (and his former supervisor). The Permanent Secretary formed a working group that used data to catalyze a sense of urgency around the issue [37]. Following a series of meetings with governors and officials in the Ministry of Public Health and other ministries, the National AIDS Committee issued a formal resolution to implement the program nationally [38]. However, progress remained slow, and the policy entrepreneur again called on his partner, the Permanent Secretary for help [37]. Following a series of meetings with governors and officials in the Ministry of Public Health and other ministries, the National AIDS Committee issued a formal resolution to implement the program nationally [38]. However, progress remained slow, and Dr. Rojanapithayakorn again called on the Permanent Secretary for help [39]. An announcement was subsequently made at a meeting of the Provincial Chief Medical Officers, requiring that they implement the program in their own provinces by the end of the following month and report back on progress within 3 months [6, 34]. Implemented fully across all provinces by mid-1992, the program played a key role in stopping the country’s HIV/AIDS epidemic in its tracks [38]. Between 1989 and 1993 condom usage in commercial sex work increased from 14 to 94% [39], with the 100% condom program causing sexually transmitted infections to fall from more than 400,000 cases per year to about 10,000, a decrease of more than 95% [6]. The scaled-up national version of the program rested on the annual distribution of 60 million free condoms to everyone [25].

Its success relied on an unusual degree of collaboration and cooperation between police, owners of sex establishments (from brothels to restaurants and tea houses to bars and nightclubs), sex workers, and public health officials and included weekly STI screenings of sex workers who were supplied with a free condoms [25, 31]. Police underwent a three-hour AIDS education training, and sex workers were empowered through the program to resist and report clients who demanded unprotected sex at brothels [33]. While the threat of brothel closure was a potent tool for non-compliance, in practice police action was rarely taken [31], though sex workers sometimes faced stigma for using condoms during casual sex [33].

By 1995, international experts estimated that Thailand’s 100% condom program helped the country avert more than two million infections [6]. The program was subsequently featured in the UNAIDS Best Practice Collection [8] and became a model for the region, with national programs starting in Cambodia, China, Myanmar, Philippines, Laos, and Mongolia, as well as faraway countries, such as the Dominican Republic [34]. UNAIDS supported similar programs in Myanmar, providing 100% of the budget, and the budget for condoms in Vietnam, while the WHO provided support to pilot program in Cambodia, Philippines, China, Mongolia, Vietnam and Lao PDR [25]. The policy entrepreneur received the Prince Mahidol Award in recognition of his HIV prevention efforts in 2009.

National uptake of the pilot program was by no means a given and faced opposition as well as a number of prominent policy alternatives. For the initial pilot project, the provincial governor served as a critical partner to the policy entrepreneur and helped bring commercial sex work operators and the police to the table for collaboration. Scaling to the national level relied critically on the evidence base the demonstration project in Ratchaburi produced and finding a receptive partner in government in the Permanent Secretary’s Office who could mandate action nationally. International partners supported further diffusion of the program within the Southeast Asian region.

### Prevention of mother-to-child transmission program

Thailand’s PMTCT program unfolded gradually. The first case of a pregnant woman with HIV reported in Thailand came in 1988 [40]. While a program providing infant formula was put in place initially, this was complemented by voluntary counselling and testing in 1993 [40] A clinical trial of zidovudine (AZT) conducted abroad had demonstrated success in reducing transmission of HIV from pregnant mothers to newborns by two-thirds in 1994 [41]. With HIV incidence rising among newborns, the WHO proposed that Thailand set up a program to prevent transmission from pregnant mothers to newborns [23]. However, access to AZT was limited in developing countries [42], and the cost of a full course was significant at $1000 and was deemed too costly for use in developing countries [41].

In Thailand’s Northern provinces, concern was growing over the number of cases of pregnant mothers with HIV, and officials started a pilot program to address the problem [23]. A four-week short course of AZT was started as a clinical trial in Bangkok in 1995 in a collaboration between the MOPH and the U.S. Center for Disease Control [40]. The short course was aimed at demonstrating effectiveness using a shorter course of medication in resource limited settings. However, as AZT effectiveness had already been demonstrated using a longer course of treatment and a standard of care established, the study raised important ethical issues [42, 43] The director of research for the Thai Red Cross sought financial support for a PMTCT program to provide pregnant mothers with HIV in Thailand the full course of AZT [42].

This critique led to brainstorming between the Red Cross and partners in the Ministry of Public Health. Despite the criticism, the trial using the placebo continued [42] and eventually concluded in 1998, achieving a 50% reduction in transmission at a tenth of the cost of the full course of treatment [41]. This level of efficacy remained, however, far below the standard established internationally with a full course of treatment [44]. While controversial, completion of the trial did however allow the country’s antenatal facilities to build more capacity to test pregnant mothers and provide antiretroviral treatment [42], effective monitoring recognized retrospectively as an important institutional precursor for successful elimination of mother-to-child transmission [45].

While this groundwork was being laid, the director of research of the Thai Red Cross, who served as a policy entrepreneur, approached a key institutional partner in the royal family about the problem of access to medication for pregnant mothers and the ethical issues raised by the current trial [42]. Princess Soamsowali agreed to provide a one million baht ($50,000) donation to the Thai Red Cross, which established a PMTCT Fund in their honor in 1996 [44]. Outside observers noted that royal involvement lent “weight to this initiative, like nothing else” [46]. The new fund also promoted the wider participation of the public by making donations tax-deductible^2^ [44]. The Red Cross approach to PMTCT using the funds aimed “for the best [standard of treatment],” rather than the short course [42]. The new program allowed hospitals to request medication and infant formula in exchange for reporting on treatment outcomes and side effects [40].^3^ The financial support not only made the program possible but provided it with some needed stability when the Asian financial crisis arrived the following year.

Following these developments, MOPH pilot projects were subsequently set up in 1997 through 1999 in partnership with the U.S. Center for Disease Control, and upon success of the pilot projects, institutional partners in the MOPH set up a national PMTCT program in 2000, which covers both public and private facilities [40]. This research on the PMTCT trials demonstrating efficacy being conducted in Thailand may have played a role in increasing the ministry’s investment in the issue [46]. Civil servants in the Upper North region initiated the project in that region [50], and when the Inspector General of that region became Director General of the Department of Health, responsible for the country’s Maternal and Child Health program [50], evidence gathered from the pilot projects in the Northern part of the country played an important role in the scale-up of the PMTCT program [51]. Lessons learned from implementation of the program in the Upper North informed national implementation [50]. Clinical trials overseen by a Red Cross HIV-NAT clinical trial unit comparing the effectiveness of different ARV regimens in infected non-pregnant adults helped keep several thousand people alive, also serving as a main driver for HIV treatment for all in Thailand under the Universal Coverage (UC) program [42].

Over time, on the basis of new scientific evidence, the drug regimen supplied by the new PMTCT Fund program evolved from one drug in 1996 to two and then three drugs in 2004 [42]. In 2000, UNAIDS included the Princess Soamsawali PMTCT Fund in its Best Practice Collection in observance of the program’s approach of mobilizing HIV funding [43]. Subsequently, MOPH programs and WHO guidelines followed the Red Cross example in using and recommending that three antiretroviral medications be used. On the basis of cost-benefit analysis, the MOPH program began to use three anti-retroviral drugs in 2010 [52], and the WHO recommended the use of three drugs that same year [42]. A fourth drug later added was found to benefit pregnant women who came very late for their PMTCT care [42].

Since 1996, more than 7000 pregnant mothers with HIV have used the PMTCT Fund [42]. Thailand’s successful national PMTCT program was built on the fact that every woman who gives birth in Thailand does so at health facilities and undergoes screening for HIV [46]. The availability of PMTCT services through both the public and private systems vis-à-vis the maternal and child health program – and inclusion of expectant pregnant migrant mothers – is something that distinguished Thailand from other countries in the region, such as Nepal and India, where the majority of births take place outside the public system [46]. Over time, hospitals began to draw on the PMTCT Fund less and less, as the same standard of treatment was provided free of charge by the MOPH, and the remaining funds were redirected to provide treatment for pregnant migrant mothers with HIV and PrEP medication for high risk men who have sex with men and transgender women [42].

WHO and UNICEF encouraged officials in the Ministry of Public Health to apply for external assessment of the program to see if it met criteria of eliminating transmission [33] The World Health Organization (WHO) certified Thailand’s elimination of mother-to-child transmission of both HIV and syphilis in June 2016 [12]. UNICEF supported dissemination first with trainings in China, Vietnam, Lao PDR, Cambodia, and Myanmar organized by Regional Office of Communicable Disease Control and provincial health offices [50]. JICA sponsored knowledge sharing events with countries in the region, such as Laos, Vietnam, and Bhutan, as well as three countries in Africa – Kenya, Tanzania, and Uganda [33]. Other regional meetings Thailand hosted shared the country’s experience with officials from Myanmar, Malaysia, China and other countries [53].

External evaluators have noted that a number of important factors that played a role in the policy’s success, including “national ownership and leadership; sustained political commitment; a favorable legal and policy environment; a well–developed national health system and the consistent strengthening of its building blocks; enhancement of community systems; and the strengthening of community interface with health systems” [40]. However, the data collected in this study point to other factors not captured in that analysis that also played an important role in the other two cases: how a policy entrepreneur used a demonstration project to provide a roadmap for national policy and the key role that partners played (in this case, the participation of Princess Soamsawali and the public) in mainstreaming policy and showing that a standard of care was possible that the national PMTCT program eventually took on as its own.

## Discussion

Our paper has offered a comparative and historical analysis of three major HIV prevention initiatives in Thailand, using inductive cross-case analysis. Our approach builds on the venerable and long-standing tradition of research on the health policy process [15, 16] – published in respected journals like *Health Policy and Planning* and *Social Science and Medicine –* and more recent emerging work in political science and sociology [17–19], using inductive analysis of comparative cases to build theory [20, 21]. We draw out the role of policy entrepreneurs as champions of reform who use demonstration projects to produce credible evidence for advocacy in order to scale up programs nationally with the help of key institutional partners. This study is informed both by relevant existing research on the different HIV initiatives, as well as interviews with key informants who worked in and around these programs in the field. However, we build on this existing work by taking a comparative and historical focus, which we believe to be a key strength of the study (see Table 1 and Table 2).

**Table 1:**
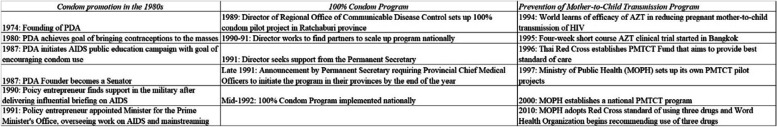
Timeline of Key Events

**Table 2:** Comparison of Cases

Case	Policy Entrepreneur	Main Concept	Results
*Condom promotion in the 1980s*	Founder of the Population and Development Association	-HIV/AID prevention -Promote the use of condoms through a humor communication strategy	Heighten visibility of condoms to prevent HIV/AIDS [2, 3, 23, 27]
*100% Condom Program*	Director of the Communicable Disease Control Regional Office	-HIV/AIDS prevention -Promote the use of condoms among high risk groups (sex workers and their clients) by distributing 60 million free condoms annually in a collaboration between police, owners of sex establishments, sex workers, and public health officials [25, 31]	A 95% reduction in new infections [6]
*PMTCT program*	Director of Research, Thai Red Cross	-Elimination of pregnant woman-to-child transmission	Reduction of the prevalence of HIV among pregnant women and of transmission from pregnant mother-to-child by 0.6 and 1.9% respectively in 2015 and eliminating PMTCT in 2016 [9, 12]

A great deal of the public health literature focuses on evaluation or analysis of single programs [9, 10, 38, 45, 54, 55]. From a certain perspective, this makes sense, as it allows for in-depth understanding of those programs. However, we argue that the lack of comparative perspective leads single case focused research to frequently miss out on a key opportunity—generating theory that helps inform broader understanding of the way the world works. That is, not just implications of the findings for a specific project but broader scientific understanding. This is in part not just a function of taking comparative approach but a disciplinary approach that puts greater emphasis on building, challenging, and refining theoretical understanding [56]. This is important, we argue, because without greater attention to the broader theoretical implications of research findings, public health science will continue to be dominated by work that presents local research findings in isolation from the broader tapestry of related work in the area and do not help challenge and refine our broader understanding of particular phenomena. We believe this study therefore complements existing research, but also helps to put findings in larger context, and pushes qualitative public health science in a direction that is more productive in relation to the aggregation of scientific knowledge from individual cases on the politics of the health policy process. Specifically, we show how and in what way demonstration projects and key institutional partnerships influence successful implementation of HIV prevention programs in three different cases. Scholarship has shown that research on global health within political science and sociology is a relatively recent phenomenon [57, 58]: this study therefore also contributes to emergent work in that area.

This study is not, however, without limitations. The research is qualitative and, as such, suffers from the usual limitations associated with qualitative research. While we were fortunate to enjoy tremendous access to high-level informants, there were still some key informants we could not interview. In addition, while we were guided by a process tracing methodology [22] and subjected our findings to review and comment by knowledgeable experts in the field in the process of creating this manuscript, our findings are ultimately constrained by our methodology and rest on our reading of the data. Additionally, as our analysis is qualitative, it is not generalizable beyond the cases. We nonetheless believe that the issues that this study draws attention to – notably the role of demonstration projects and key institutional partners – point to neglected issues in the literature and advance our theoretical understanding of phenomena that influence success in HIV prevention, building on earlier referenced work in new and important ways. In this way, our paper is a call for researchers to think about the phenomena of demonstration projects and institutional partnerships not in isolation but in relation to demonstration projects and institutional partnerships more broadly.

## Conclusion

This article has explored how committed policy entrepreneurs deepened institutionalization of Thailand’s HIV/AIDS prevention programs in three different cases: condom promotion by a civil society organization, the 100% condom program, and the national PMTCT program. In all three cases, policy entrepreneurs succeeded in mainstreaming innovative approaches to prevention into national HIV/AIDS policy while initially occupying positions on the periphery of power – as founder of a civil society organization in the country’s Northern most province, as a civil servant working in a province outside of Bangkok, and as director of a non-profit research center.

Our inductive cross-case analysis draws out the role that demonstration projects play in producing credible evidence that can be used to advocate for policy adoption and the role that key institutional partners played in helping to elevate and mainstream policy entrepreneurs’ ideas, so that they could be scaled nationally. In the case of the PDA’s condom promotion efforts, a norm promoted by a civil society organization based in the country’s Northern most province around safe sex was first taken up within the military and later incorporated into national HIV/AIDS prevention efforts. In the 100% Condom Program case, a pilot program started by a ministerial official that met with success in a province outside of Bangkok, with the help of partners in government and the police, was implemented nationally with the support of receptive officials in the national ministry. In the PMTCT program case, a demonstration program started by the Red Cross with support from the royal family and the public laid the basis for what the emerging national PMTCT program could become. Policy entrepreneurs’ success was contingent on identifying and taking advantage of political opportunities that were available in the different historical periods and required them to draw on their social capital in order to overcome obstacles.

## Supplementary Information

**Additional file 1: ** Interview Protocol.

## Data Availability

Data used for this paper is available from the corresponding author on reasonable request.

## Abbreviations

AZT: Zidovudine
HIV/AIDS: Human Immunodeficiency Virus / Acquired Immunodeficiency Syndrome
MOPH: Ministry of Public Health
NGO: Non-Governmental Organization
OECD: Organization of Economic Cooperation and Development
PDA: Population and Development Association
PMTCT: Prevention of Mother to Child Transmission
STI: Sexually Transmitted Infection
WHO: World Health Organization

## Footnotes

1 Although only an informal group, this group, called the Sampran Forum, has met monthly since 1986, taken up a wide range of issues, and has had important impact on a number of different policy issues [21, 35, 36].

2 Recent work has pointed to the need to examine the way in which tax policy plays an important role in shaping health policy [47].

3 This was not the first time in which medical professionals had relied on royal patronage for pilot medical projects in areas that were critical to human life; royal support also played an important role in expanding access to the Hepatitis B vaccine [48], which was adopted as policy in Thailand in 1988 and incorporated into the national immunization program in 1992 [49].
