# Supplementary material for: Common factors in HIV/AIDS prevention success: lessons from Thailand
Source: BMC Health Serv Res. 2022 Dec 6;22:1487. doi: 10.1186/s12913-022-08786-6 (PMC9727911; doi:10.1186/s12913-022-08786-6)
Supplement: Supplementary file 1 — Additional file 1: Interview Protocol. [file 12913_2022_8786_MOESM1_ESM.doc]

**Interview Protocol**

1. Introduction
2. Overview of Oral Informed Consent Declaration and Agreement
3. Semi-structured, Open-Ended Interview Questions Basic Sample Script (interviews may include related follow-up questions that probe respondent answers)

Who is responsible for creating Thailand’s X policy? How did the policy come about?

What are/were the key features of the policy?

Which organizations, institutions, and/or publications recognized it internationally?

Why did Thailand’s X policy achieve so much recognition?

Are there other countries that emulated Thailand’s policy model? Which countries?

How did X country/ies learn about that policy? What was critical in enabling that knowledge to travel abroad?

Did Thai officials play any role in helping to disseminate knowledge about the model? If so, what did they do? How did they do it?

If Thai officials did not play any or much of a role in helpful to disseminate knowledge about the policy, how did other countries learn about the policy?

Were there competing policy models in this area? What were the other models? What were their key features?

Did the Thai policy models achieve more notoriety than the other models? By what measure?

Which international organizations and other journals/organizations/people helped to popularize knowledge of the Thai model?

Are there any [social or cultural, organizational, political, etc] factors that help to explain how and why it is that Thailand was able to produce so many model public health policies, such as X, X, X, etc? What are they?

Do these factors help explain policy success in all the policy areas under study, or just some of them? Why is that?

Are there any [social or cultural, organizational, political, etc] factors that help to explain how and why it is that Thailand’s policies were recognized internationally in so many different policy areas, such as X, X, X, etc? What are they?

Do these factors help explain international recognition and policy diffusion in all the policy areas under study, or just some of them? Why is that?

1. Wrap-Up and Thanks for Interview
